# Correction: A protocol for using human genetic data to identify circulating protein level changes that are the causal consequence of cancer processes

**DOI:** 10.1371/journal.pone.0352426

**Published:** 2026-06-23

**Authors:** Lisa M. Hobson, Richard M. Martin, Karl Smith-Byrne, George Davey Smith, Gibran Hemani, Joseph H. Gilbody, James Yarmolinsky, Sarah E. R. Bailey, Lucy J. Goudswaard, Philip C. Haycock

There are errors in the Funding statement. The correct Funding statement is as follows: LMH is supported in part by grant MR/W006308/1 for the GW4 BIOMED MRC DTP, awarded to the Universities of Bath, Bristol, Cardiff and Exeter from the Medical Research Council (MRC)/UKRI. RMM, LJG and PCH are supported by a Cancer Research UK 25 (C18281/A29019) programme grant (the Integrative Cancer Epidemiology Programme). RMM is also supported by the NIHR Bristol Biomedical Research Centre which is funded by the NIHR (BRC-1215-20011) and is a partnership between University Hospitals Bristol and Weston NHS Foundation Trust and the University of Bristol. Department of Health and Social Care disclaimer: The views expressed are those of the author(s) and not necessarily those of the NHS, the NIHR or the Department of Health and Social Care. SERB was supported by an NIHR Advanced Fellowship (NIHR 301666) whilst undertaking this work. Additional support was provided by the Higgins family. The funders had no role in study design, data collection and analysis, decision to publish, or preparation of the manuscript.
